# Monoclonal Antibodies in Preclinical EAE Models of Multiple Sclerosis: A Systematic Review

**DOI:** 10.3390/ijms18091992

**Published:** 2017-09-16

**Authors:** Katja Schmitz, Gerd Geisslinger, Irmgard Tegeder

**Affiliations:** Institute of Clinical Pharmacology, University Hospital Frankfurt, 60590 Frankfurt, Germany; Schmitz@med.uni-frankfurt.de (K.S.); geisslinger@em.uni-frankfurt.de (G.G.)

**Keywords:** autoimmune encephalomyelitis, monoclonal antibody, immune system, multiple sclerosis

## Abstract

Monoclonal antibodies (mAb) are promising therapeutics in multiple sclerosis and multiple new candidates have been developed, hence increasing the need for some agreement for preclinical mAb studies. We systematically analyzed publications of experimental autoimmune encephalomyelitis (EAE) studies showing effects of monoclonal antibodies. A PubMed search retrieved 570 records, out of which 122 studies with 253 experiments were eligible based on experimental design, number of animals and presentation of time courses of EAE scores. Analysis of EAE models, treatment schedules, single and total doses, routes of administration, and onset of treatment from pre-immunization up to 35 days after immunization revealed high heterogeneity. Total doses ranged from 0.1 to 360 mg/kg for observation times of up to 35 days after immunization. About half of experiments (142/253) used total doses of 10–70 mg/kg. Employing this range, we tested anti-Itga4 as a reference mAb at varying schedules and got no, mild or substantial EAE-score reductions, depending on the mouse strain and onset of the treatment. The result agrees with the range of outcomes achieved in 10 reported anti-Itga4 experiments. Studies comparing low and high doses of various mAbs or early vs. late onset of treatment did not reveal dose-effect or timing-effect associations, with a tendency towards better outcomes with preventive treatments starting within the first week after immunization. The systematic comparison allows for extraction of some “common” design characteristics, which may be helpful to further assess the efficacy of mAbs and role of specific targets in preclinical models of multiple sclerosis.

## 1. Introduction

Monoclonal antibodies are increasingly used and developed as therapeutics in multiple sclerosis (MS). Prominent candidates are natalizumab targeting α-4 integrin and two “old” antibodies against lymphocyte surface markers, alemtuzumab (CD52) and rituximab (CD20), which have been repurposed for multiple sclerosis, and the recently from the U.S. Food and Drug Administration (FDA)-approved ocrelizumab, also targeting the B-cell antigen, CD20 and is the first for relapsing remitting (RRMS) and primary progressive forms of MS (PPMS) [1,2]. The success with these monoclonals in MS has raised the scientific and pharmaceutical interest to develop additional, better or less problematic mAbs, and several new candidates are being tested in phase-2 or -3 clinical trials. About one third of putative novel MS therapeutics are monoclonal antibodies [3]. Natalizumab, which was approved in 2004, is a second line drug despite its high efficacy because unfortunately, it is associated with occurrence of progressive multifocal leukoencephalopathy, a serious virus infection with about 20% mortality [4]. Alemtuzumab targets the CD52-antigen on the surface of mature lymphocytes, monocytes, dendritic cells and granulocytes and has a long history in the treatment of some types of leukemia. It was reintroduced under a novel trade name for MS in 2014 [5], followed by daclizumab in 2016, which is a humanized IgG1 mAb blocking receptor binding of interleukin (IL)-2 to CD25, previously used for prevention of kidney transplant rejection. Alemtuzumab rapidly leads to clinical and radiographic remission of MS but it is associated with the risk of developing new autoimmune disorders [6]. Recently, ocrelizumab was found not only to reduce the relapse rate in RRMS [1], but to reduce also the disease progression in PPMS [2]. Presently, mAbs are second line drugs for escalation therapy mostly for relapsing-remitting MS, but the success strongly suggests that mAbs targeting immune cell subtypes, surface antigens or their ability to penetrate the blood brain barrier specifically interfere with the autoimmune attack that leads to a destruction of the myelin sheaths in MS. A number of targets are being evaluated, in particular CD40 [7,8] and its ligand and other tumor necrosis factor (TNF) family members [9,10,11] and antibodies targeting IL-12 [12], IL-17 [13] or IL-21 [14].

To assess the efficacy of novel MS-specific mAbs, and to further repurpose monoclonal antibodies, they have to be tested in terms of efficacy and safety in pre-clinical models, raising the need for some agreement about experimental settings and study designs to increase comparability and predictability for the efficacy in humans. Experimental autoimmune encephalomyelitis (EAE) is the most common animal model for MS, but not beyond dispute because of the differences of human MS and rodent EAE [15,16,17]. This limitation applies to all models, no matter whether the relapsing remitting EAE (RR-EAE) in SJL/J and ABH Biozzi mice or Dark Agouti rats, or the primary progressive EAE (PP-EAE) in C57Bl6 mice, or the monophasic EAE in Lewis rats are used [18]. This is a major challenge for all candidate drugs, but specifically for antibodies, because some targets may need to be humanized owing to low homology [19,20]. An example is rituximab, which is targeting the B-cell antigen, CD20 with 75% homology between mice and humans. It is quite effective in human MS [21], failed in the C57BL6 mouse but strongly suppressed EAE in a human CD20 transgenic mouse on a C57BL6 genetic background [19] suggesting that high specificity for the human protein may preclude efficacy in the mouse unless the target is humanized. The results with common EAE models, which mainly rely on T-cells further suggests that models are needed, which include B-cells, such as some spontaneous EAE models, like the TCR1640 transgenic mice [22]. However, heterogeneity of the clinical courses in TCR1640 mice limit their usefulness for drug evaluation studies. To solve this EAE dilemma, marmoset monkeys are increasingly used including therapeutic studies with mAbs, but mostly with highly variable effects, which mainly consisted in a right shift of the onset of clinical symptoms, without affecting disease incidence or severity after discontinuation of mAb treatment [10,23,24,25]. In addition to EAE, cuprizone-evoked reversible demyelination is used to assess some aspects of de- and remyelination, but effects of cuprizone are not mediated by autoimmune attack and hence, mechanistically different. Consequently, only one study so far shows at the histology-level that a mAbs might improve remyelination [26].

Because of the shortcomings of EAE models, there is some agreement among EAE researches to test novel candidates in at least two different models, because the predictive value increases with the number of models in which efficacy can be demonstrated. Mostly drugs are continuously or once daily administered during the course of the EAE disease and a crucial decision is when to start the therapy, for example at onset or peak of clinical symptoms or shortly after immunization or during intervals. Above these considerations, which also apply for mAbs, the latter are very versatile in terms of the potential dosing regimens or schedules. Even in humans, monoclonal antibodies may be administered every 4 weeks or at intervals of 6 months, and mostly no longer than 2 years because longer treatments increase the risk of viral infections. These human schedules cannot be re-translated directly to the mouse so that there is presently no obvious rationale for choosing a specific regimen in a rodent EAE study.

To address the current uncertainty in terms of preclinical study designs for evaluation of mAbs in EAE models, the present systematic review summarizes results of various mAb-EAE studies in mice, rats and marmosets ranging from 1990–2017 and provides a searchable Excel spreadsheet detailing designs and schedules. The review extracts some agreeable design strategies, supported by experimental data for anti-Itga4.

## 2. Results

### 2.1. Paper Evaluation

**Figure 1:**
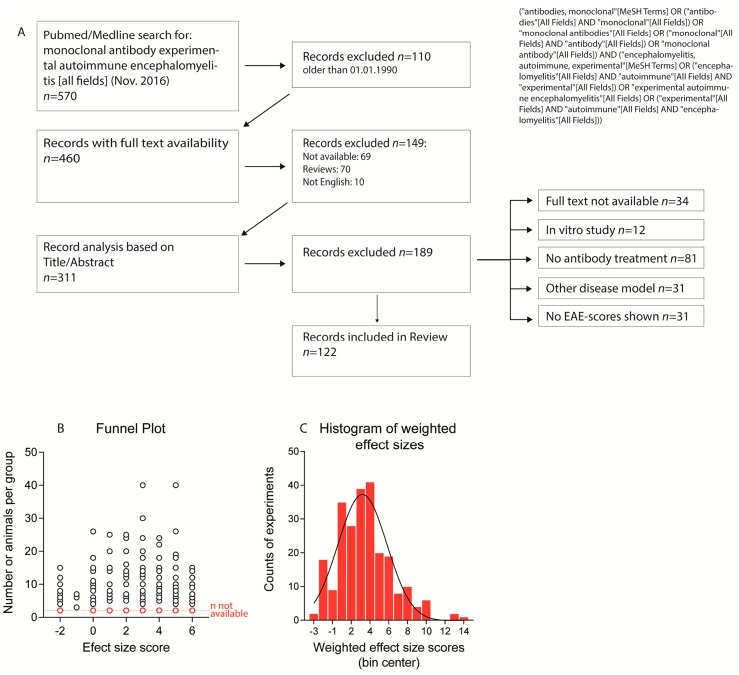
(**A**) Flow diagram showing the strategy of the literature search and paper selection criteria; (**B**) Funnel plot showing the number of animals per experiment vs. effect sizes. Experiments for which *n*-numbers were not reported are marked in red and presented below the other experiments; (**C**) Histogram showing the frequency of weighted effect sizes of experiments suggesting a Gauss distribution.

Overall, 122 studies were eligible based on the selection criteria (Figure 1A, Table S1). They assessed monoclonal Abs against 78 different targets in 253 experiments. All studies except one [27] provided graphical presentations of time courses of the clinical score. Marmoset studies presented results as individual time courses because of low numbers (4–6 per group) and high inter-individual variability [10,24,25,28]. The marmoset studies were mostly underpowered for testing differences in EAE severity. Instead, the disease onset was used as an indicator of therapeutic efficacy. For 3 studies, information about the doses was missing [29,30,31]. The number of animals per treatment group ranged from 3 to 40 with a mean of 9.6–11.3 for lower and higher margins of group sizes. Sixty-eight experiments in 34 studies were done with <6 animals per group and numbers were not available for 13 studies involving 24 experiments.

Although EAE scores are ordinal-scaled data, several studies used parametric statistical tests. Nineteen studies employed unpaired Student’s *t*-tests for comparison of the clinical scores between treatment groups, either for each time point individually, or by using the cumulative scores or without further information. Further 23 studies used ANOVAs to compare treatment effects either using one-way ANOVA for area under the curves (AUCs), or two-way ANOVAs for “time” by “group”. In 47 studies, the non-parametric Mann Whitney U, Kruskal Wallis or Wilcoxon tests were employed. Contingency tables for EAE incidence were presented in 11 studies using either χ2 statistics of Fisher’s exact test and 27 studies did not employ statistical methods or did not report on the type of statistics used. 

Several studies presented the mean or median time course of EAE scores without indices of inter-variability and the majority of the studies did not present sufficient data to calculate the effect size according to Cohen’s D. Therefore, we used an estimate based on the graphical presentation of the EAE time courses. Because variances were not available, the number of animals per treatment group was used for weighting of the effect sizes. Funnel plots did not reveal a bias towards publication of positive effects (Figure 1B) and the frequency distribution of the weighted effect size scores followed a normal distribution (Figure 1C). “No-effect” results (33 experiments in 25 studies) were mostly reported in papers, which also presented experiments where attenuation or aggravation was observed. These studies mostly provided plausible explanations for failure in a specific experiment, such as too low [9] or too high dose [32], too early [33] or too late treatment onset [34], different antibody clones [11], different EAE models [35] or dual functions of the targets [36].

### 2.2. Treatments Schedules and Doses

**Figure 2:**
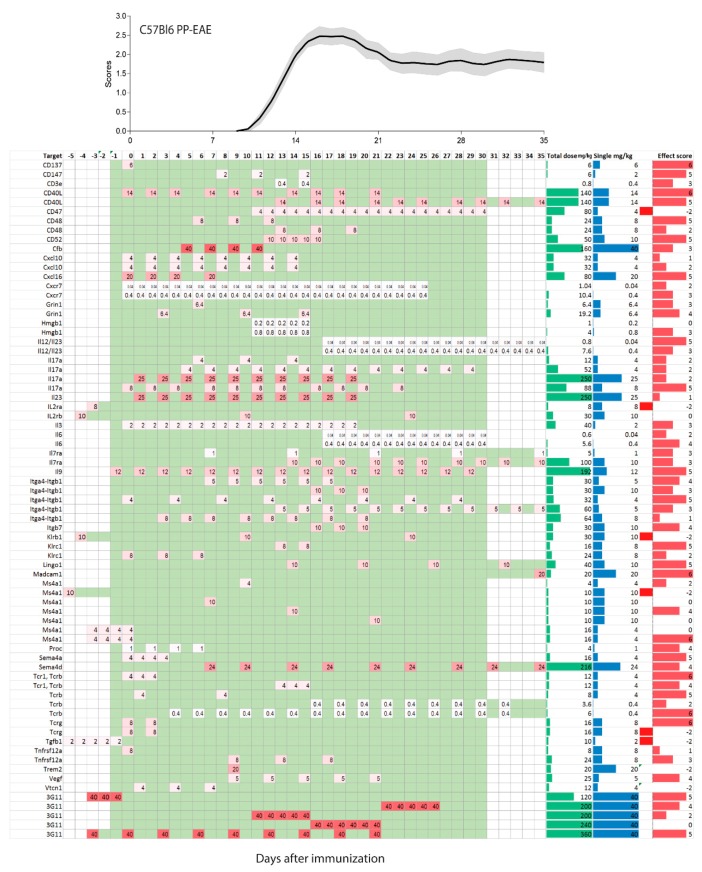
Treatment schedules with monoclonal antibodies in the C57BL6 mouse model of primary progressive experimental autoimmune encephalomyelitis (PP-EAE) after active immunization on day zero. The schedules are aligned with the mean time course ±95% confidence interval (red area) of the pooled clinical experimental autoimmune encephalomyelitis (EAE) scores of 92 mice of our own studies. Mice were untreated or receiving placebo. The left panel shows the targets, the right columns show the total dose in mg/kg (green), single dose (mg/kg, blue) and the effect size score (red), not weighted fro number of mice. The effect sizes range from 6 (very strong reduction of EAE scores) to 0 (no effect), and is negative (−1 or −2) where the antibody made the disease worse. The schedule ranges from −5 days up to 35 days and is color-coded. The darker the red, the higher was the single dose. Days without treatment are green, days without observation white. Observations > 35 days in 2 studies were cut.

**Figure 3:**
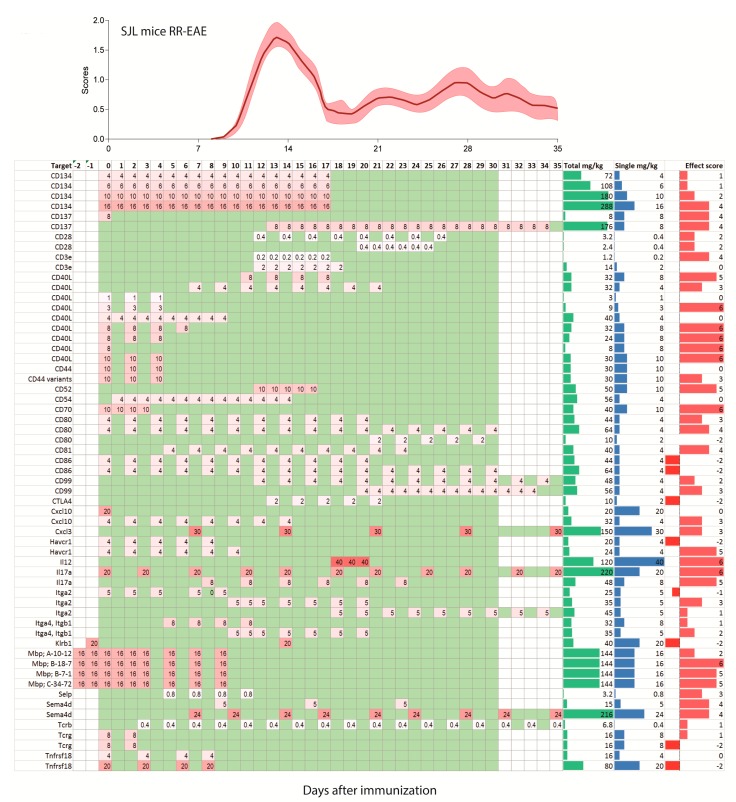
Treatment schedules with monoclonal antibodies in the SJL/J mouse model of relapsing-remitting EAE (RR-EAE) after active immunization on day zero. The schedules are aligned with the mean pooled EAE time course ±95% confidence interval (red area) of 112 mice of our own studies. Mice were untreated or receiving placebo. The schedules range from −3 days up to 35 days and are color-coded as in Figure 2. The darker the red, the higher was the single dose. Days without treatment are green, days without observation white. Two schedules with observation times > 35 days were cut.

**Figure 4:**
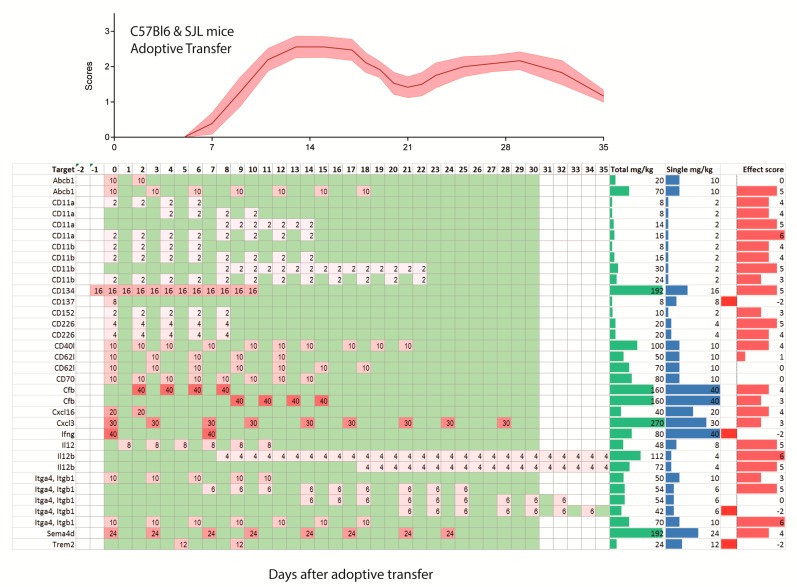
Treatment schedules with monoclonal antibodies in the C57BL6 mice (upper 5 rows) and SJL/J mice after induction of EAE by adoptive transfer of auto-aggressive immune cells obtained from actively immunized mice. The illustration of the time course of the clinical scores after adoptive transfer is based on the literature. The schedule ranges from −2 days before adoptive transfer up to 35 days and is color-coded as in Figure 2. The darker the red, the higher was the single dose. Days without treatment are green, days without observation white. Two schedules with observation times > 35 days were cut.

**Figure 5:**
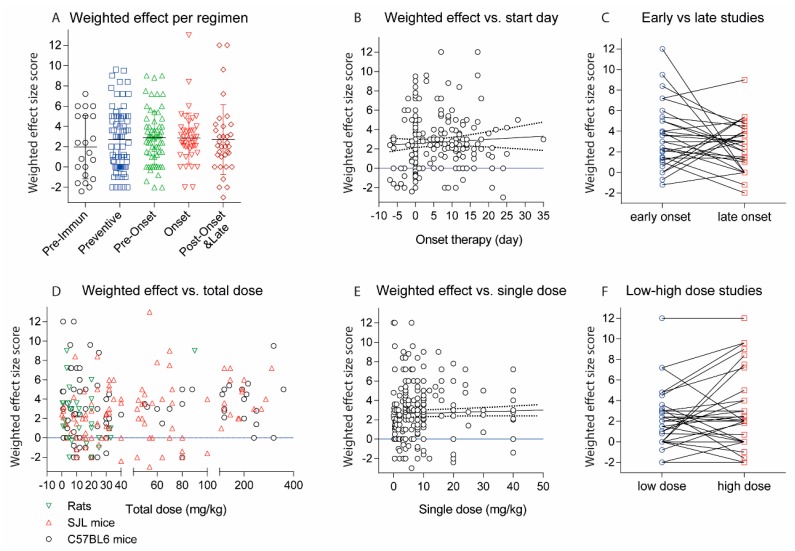

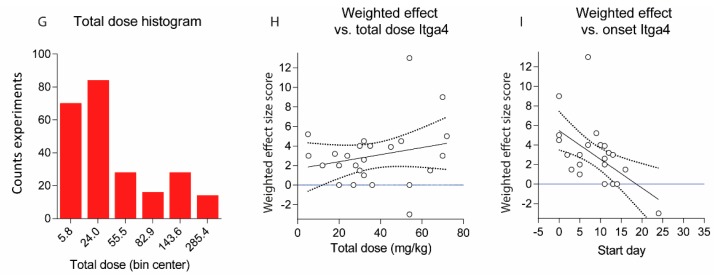
(**A**) Scatter plots showing the weighted effect size scores of experiments with different treatment regimens. The effect size score ranges from 6 (very strong reduction of EAE scores) to 0 (no effect) and is negative (−1 or −2) if the antibody aggravated the disease. The scores were weighted according to the number of animals per treatment group (*n*) by multiplication of the effect size with *n*/10; (**B**) Association of weighted effect size scores with the day of onset of the treatment. The line shows the linear regression plus 95% CI (dotted lines), not significant (n.s); (**C**) Weighted effect size scores for studies, which directly compared early (preventive or pre-onset) and late (onset, post-onset and late) therapeutic regimens. For three different schedules, the earliest and latest were used. For studies with 4 schedules 2 pairs were used. There was no association between onset of therapy with the effect size, Wilcoxon matched-pairs signed rank test, *p* = 0.2406; (**D**) Association of weighted effect size scores with the total dose in mg/kg for C57Bl6 and SJL/J mice and rats. Mice with mixed backgrounds are included. If the dose was given in µg per animal, the amount in mg/kg was estimated based on a body weight of 25 g per mouse, 300 g per Lewis rat and 200 g per dark agouti (DA) rat; (**E**) Association of weighted effect size scores with the single dose in mg/kg. The line shows the linear regression plus 95% CI (dotted lines), n.s; (**F**) Weighted effect size scores for studies, which directly compared 2 or more different doses. For studies with 3 doses the lowest and highest were used. Each line represents one study. Overall, there was no dose-effect relationship, Wilcoxon matched-pairs signed rank test, *p* = 0.4493; (**G**) Histogram of total doses in mg/kg including all experiments; (**H**) Association of weighted effect size scores with the total dose in mg/kg for experiments targeting VLA-4 (Itga4/Itga2 dimer) with Itga4 antibodies. The line shows the linear regression plus 95% CI (dotted lines), n.s; (**I**) Association of weighted effect size scores with the day of treatment onset for experiments targeting VLA-4 (Itga4/Itga2 dimer). The line shows the linear regression plus 95% CI (dotted lines). The slope of the regression line differed significantly from zero, *p* = 0.0046.

In humans, single doses of monoclonal antibodies in MS patients range from 1–2 mg/kg for daclizumab, 3–6 mg/kg for natalizumab, 1, 3 or 10 mg/kg for alemtuzumab and 10–15 mg/kg for rituximab. Hence, translated to mice, one may consider a single dose of 1–15 mg/kg as a reasonable start, and most studies used doses in this range (92 studies with 181 experiments). There was much less consensus in terms of the dosing frequency, intervals and start of treatment. Particularly the latter ranges from 7 days before immunization [33,37,38] up to 35 days after active immunization [39]. The heterogeneity of treatment schedules is illustrated in Figure 2, Figure 3 and Figure 4 for PP-EEA in C57Bl6 mice, RR-EAE in SJL/J mice and for adoptive transfer models, respectively. Twenty-eight studies compared effects of different treatment regimens (Figure 5A), but in contrast to small molecular MS drugs, which normally show stronger efficacy on preventive (i.e., early) therapy, there was no significant association between onset of therapy and observed efficacy (Figure 5A–C). Instead, experiments employing pre-immunization or pre-adoptive transfer regimens had a high rate of unfavorable worsening outcomes (8 out of 30 experiments), which could not be explained by targeting protective proteins or cells.

Most studies used dosing intervals of 1–3 days (Figure 2, Figure 3 and Figure 4), but the number of doses ranged from single injections in 34 experiments (22 studies) up to 30 doses (2 studies) [12,23]. Hence, total doses ranged from 0.1 to 360 mg/kg for an observation time of 35 days after immunization or adoptive transfer (Figure 5D–G). Twenty-five studies assessed efficacy at two or more different total doses (Figure 5F). Overall, there was no association between total or single doses with weighted effect sizes (Figure 5D–F). The result was similar with and without inclusion of “aggravation-experiments”. In studies where the lower dose was less effective (5 studies) it was 10–100-fold lower than the “common” doses, hence rather a placebo. Nine studies testing anti-Itga4 in 21 experiments did not show dose-dependent effects but revealed that the efficacy dropped if treatment was initiated after onset of the clinical symptoms (Figure 5H,I).

### 2.3. Natalizumab Effects Depending on Treatment Schedules

**Figure 6:**
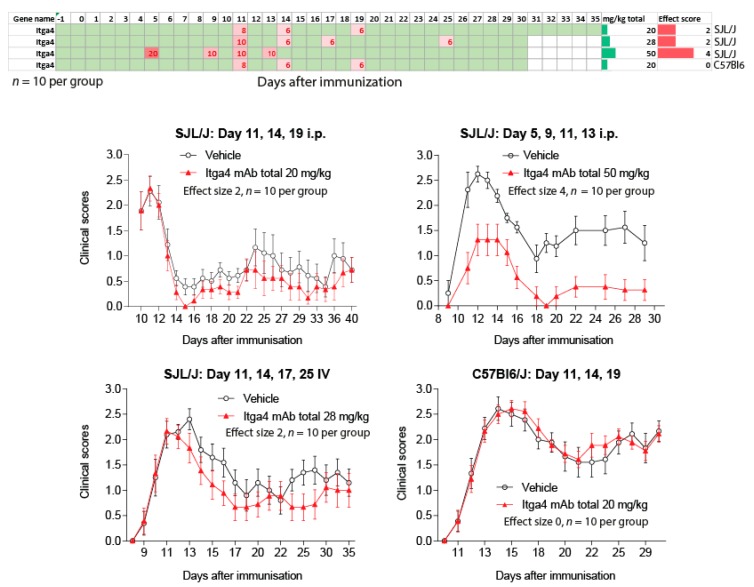
Time courses of EAE scores in SJL/J or C57BL6 mice treated with anti-Itga4 or placebo according to the schedules and doses presented in the figure. The single doses ranged from 6–20 mg/kg. Each experiment comprised 10 mice per treatment group and data are the mean ± sem (standard error of mean). The effect size scores in SJL/J was 2 (mild reduction) to 4 (substantial). There was no effect in C57BL6 mice. The top panel shows the schedules.

To address the impact of treatment schedules relative to disease onset we assessed the effects of anti-Itga4 in four independent experiments (Figure 6), testing two routes of administration (intraperitoneal (i.p.) vs. intravenous (IV)), two strains (SJL/J, C57BL6) and two schedules (peak vs. pre-onset). In 2 experiments with SJL/J mice we observed a mild reduction of EAE scores, either with i.p. or IV injections, starting at the time of the first peak (Effect size 2, onset day 11). With the same i.p. schedule, anti-Itga4 had no effect in C57BL6 mice. However, anti-Itga4 considerably reduced EAE scores (effect size score 4) in another experiment in SJL/J mice, in which treatment was initiated before onset of the scores 5 days after immunization (preventive), hence supporting the idea that efficacy of anti-Itga4 depended on the day of treatment start relative to the onset of clinical scores.

### 2.4. Targets: Favorable Candidates

**Figure 7:**
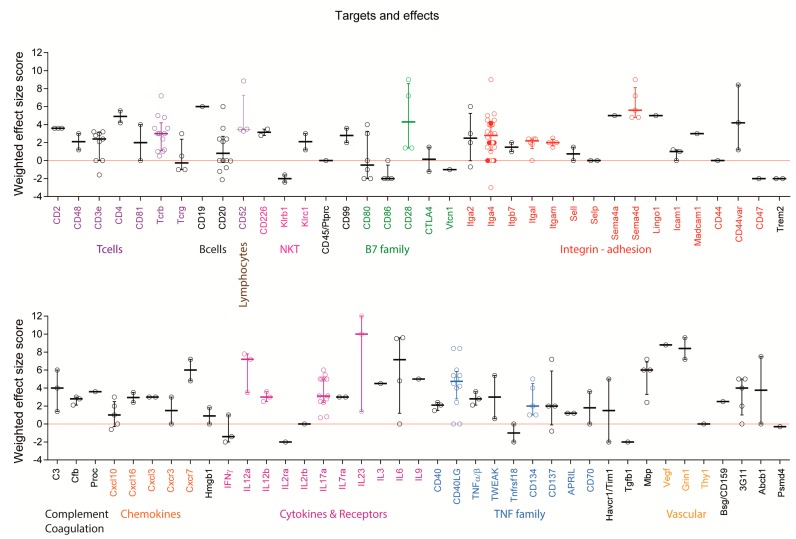
Scatter plots showing the weighted effect size scores per experiment including targets with at least 2 experiments. Itga4 (VLA-4) was the most frequently targeted molecule. Results of our own studies targeting Itga4 appear as filled red dots. Worsening with anti-Itga4 occurred in a late adoptive transfer experiment, where it increased the relapse rate. Promising targets with at least 4 positive experiments (effect size > 0) and no aggravations are highlighted by color of the respective group. For some targets, the classification of “promising” is based on results of only one study (3G11 epitope, Mbp, CD134/Tnfrsf4, Itgal/CD11a). One failure (effect size 0) for CD40LG was caused by low dose. For Ms4a1/ani-CD20 worsening was caused by B-cell depletion pre-immunization.

**Figure 8:**
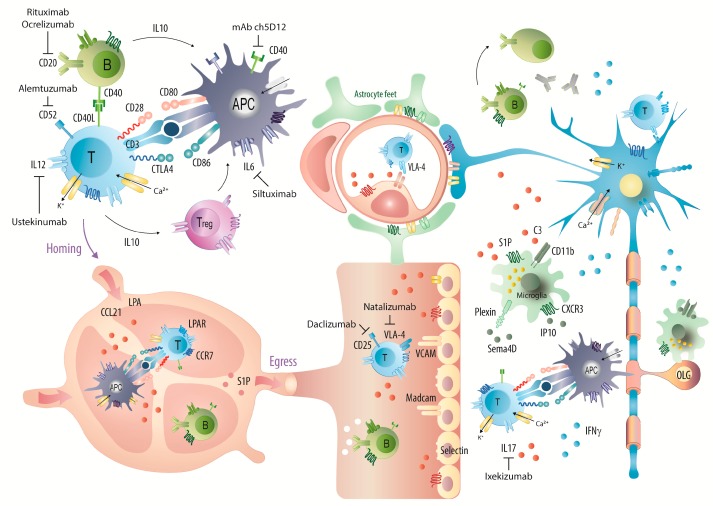
Graphical illustration of the majority of candidates, which were targeted with monoclonal antibodies in EAE studies and mechanisms of some approved mAbs or promising candidates. Abbreviations: APC, antigen presenting cells; CD, cluster of differentiation; CCR and CXCR, chemokine receptors; CCL21 and IP10, chemokines; IFN, interferon; IL, interleukin; LPA, lysophosphatidic acid; OLG, oligodendrocytes; S1P, sphingosine-1-phosphate; VLA-4, very late antigen 4; VCAM, vascular cell adhesion molecule. Mechanisms of currently approved disease modifying drugs in MS have been illustrated in [74].

Monoclonal antibodies were directed against 78 different targets (Figure 7 and Figure 8) including cell surface markers of T cells, B cells, dendritic cells (DCs), microglia and macrophages, endothelial cells and NK cells and secreted factors including cytokines, chemokines, semaphorins and complement factors. Failures appeared to occur randomly in some experiments with several targets, which were tested in more than one study including anti-Itga4, which failed in 2 experiments [40,41]. The same antibody, PS/2, provided moderate to strong EAE reduction in other studies at similar doses [40,41,42,43,44] and aggravated symptoms in one study [40], the latter in an adoptive transfer experiment with late onset of therapy, where the mAb increased the relapse rate. Similarly, anti-CD40L failed twice [9,45], while the same antibody (MR-1) was moderately to strongly effective in other studies, all using SJL/J mice with the same preventive regimen and similar doses [7,8,9,11,46]. One failure with anti-CD40L was likely caused by a low dose, the other without obvious reason. Nevertheless, CD40L or its receptor appear to be promising targets. Other promising candidates with at least 4 positive experiments (effect size > 0, highlighted by color lettering in Figure 7) and no aggravations, include Sema4d [30,47], CD28 [48,49,50], CD52 [51,52,53], IL-12 [12,25,46,54], IL-17 [13,28,55,56,57,58] and some TNF family members [59]. Experiments showing strong efficacy of mAbs targeting Itgal/CD11a and Itgam/CD11b were obtained all in one study using 4-6 mice per group [60]. In addition, some mAbs were so far tested only once, such as anti Lingo1 [61], but might be promising candidates.

### 2.5. Targets: Unfavorable Candidates

Monoclonal antibody treatments aggravated EAE symptoms in 30 experiments of 26 studies (Figure 5A). For some targets, aggravation depended on the treatment schedule, and overall aggravation tended to be more frequent in pre-immunization or preventive studies as compared to treatments starting at or after onset (Figure 5A). For example, pre-immunization treatment with anti-Ms4a1/CD20 aggravated the disease [31,33,38] whereas later treatment reduced EAE scores [33] or had no effects [38] suggesting that pre-immunization B cell depletion favored the auto-aggressive T-cell response. Indeed, pre-immunization depletion of B cells reduced the numbers of regulatory B and T cells (Tregs) [33], which are essential to control the autoimmune response. Targeting the Treg surface antigen, CD25/IL2ra similarly aggravated the disease [62].

Targeting of natural killer cell surface molecules caused dual effects. Blocking Klrb1, which likely confers inhibitory functions on natural killer T (NKT) cells aggravated EAE symptoms [63], whereas blocking Klrc1 [64] reduced the scores. Worsening also occurred by targeting CD86 [36,65], which is expressed on antigen-presenting cells (APCs) and provides costimulatory signals necessary for T cell activation and survival. It is the receptor for two different proteins on the T cell surface, CD28 for activation and CTLA4 for attenuation, and it works in tandem with CD80 to prime T cells. They are all B-7 family members. In this context, blocking CD80 or CTLA4 had dual effects on EAE scores [36,65,66], whereas blocking CD28 attenuated the disease [48,49]. Hence, outcomes were partly but not entirely explained by the different functions of the players. Further unfavorable outcomes were observed in experiments employing mAbs against interferon (IFN) [67,68] or the cell surface proteins Tim1/Havcr1 [69] and Trem2 [70]. The latter have co-stimulatory, pro-inflammatory functions and soluble Trem2 is increased in cerebrospinal fluid (CSF) of MS patients [71]. Hence, it is not clear why the respective mAbs not only missed the expected therapeutic benefit but aggravated the symptoms.

### 2.6. Experimental Autoimmune Encephalomyelitis (EAE) Models and Strains or Species Effects

Unlike other MS drugs, efficacy of monoclonal antibodies did not substantially differ between C57BL6 and SJL/J mice in studies, in which both strains were directly compared. Seventeen studies compared two or more different strains or active immunization vs. adoptive transfer. The outcomes were similar, except for anti-CD70 and anti-CD137, which failed or aggravated symptoms on adoptive transfer (effect size 0 or −2) but completely prevented or strongly reduced EAE on active immunization (effect size 6 or 4–5) [35,72]. Inversely, anti-CXCR3 was strongly effective on adoptive transfer, but failed after active immunization [73]. Only one study addressing Sema4d directly compared mice and DA rats with similar outcomes [47]. All other studies with rats used the monophasic model in Lewis rats (15 with 33 experiments) but with multiple methods to evoke the disease, 20 experiments with active immunization, 13 with adoptive transfer. The total dose ranged from 0.3–30 mg/kg and the regimens were preventive or pre-onset owing to the monophasic course in this model. Hence, studies using rats were even more diverse.

## 3. Discussion

The present compilation of EAE studies that tested mAbs illustrates the heterogeneity of treatment schedules, dosages, models and outcomes and reflects the uncertainty in terms of study design, which may be most qualified to reveal the impact of the target rather than that of timing and dosing. The heterogeneity of the studies reflects the EAE dilemma, which is the best model for human MS but cannot be translated 1:1 to human MS, neither in terms of mechanisms nor therapeutic success [15,16,17]. Obviously, there is no universally valid approach but comparison of the studies and own results provides some general “rules”, which may be useful for future studies: (1) Intravenous injection had no advantage over intraperitoneal injections, supported by a number of studies showing bioavailability of the antibody after i.p. injection [47]. Oral treatment may have an impact on the disease, but by changing immune balances in the gut [32,75], because systemic bioavailability by this route was not observed; (2) Start of treatment before immunization had no advantage and was rather associated with failure; (3) Start of treatment before onset of clinical symptoms (preventive) improved the outcome for some antibodies, but overall there was no obvious advantage, likely because cells or soluble factors must be abundant and available in the blood for full exploitation of the neutralizing effects. In addition, too early treatment may elicit counterbalancing adaptations that limit the antibody efficacy. Unfortunately, very few studies tested the in vivo time course of target neutralization or cell depletion after i.p. or intravenous administration of the respective antibody. Mostly, such data are available for B cell depletion after injection of antibodies targeting B cell surface antigens [19,38,76], but rarely for soluble factors [47]; (4) Short-term treatment over 3–4 days was equally effective as long-term treatment with injections every day or every other day; (5) A reasonable range of 1–10 mg/kg may be suggested as a single dose and 10–70 mg/kg as the total dose up to 35 days after immunization. Fifty percent of the experiments (142 out of 253) fall into this range; (6) Depending on the model, observation times of 30–35 days are required to avoid overseeing of late effects. For example, one study observed a higher rate of late relapses in mice treated with anti-Itga4 in an adoptive transfer EAE model [40], which would have been missed with shorter observation.

Correct timing of treatment and monitoring are crucial. Particularly, for mAbs targeting autoimmune B cells, opposing effects were observed with early and later treatments, because different B cell subtypes promote or inhibit EAE pathophysiology [33,77]. Particularly, spontaneous EAE models rely in part on B cells [22,41], but were used in only one study with anti-Itga4 [41]. Memory B cells, which cross the blood–brain barrier, are believed to undergo re-stimulation, antigen-driven maturation, clonal expansion, and differentiation into antibody-secreting plasma cells within the central nervous system. Hence, mAb treatment targeting B cells must prevent the entry or the local expansion without interfering with their regulatory effects. This also holds true for T cells and APCs and may explain the duality of effects of mAbs targeting B-7 family members. However, some outcomes remained enigmatic and question the reliability and reproducibility, particularly if groups comprised <8 animals. Small n-numbers applied to about half of the experiments (116 out of 253).

For quantitative comparisons and regression analyses, we used an estimate of the effect size based on the graphical presentation of the time courses of the EAE scores, body weights where available, and the change of the AUC under mAb treatment. Although the effect size scores were independently extracted by two researchers with high agreement (±1 of the effect size score), and without knowledge of the interpretation of the result by the respective authors, the estimate is an approximation of the outcome and might miss details, which may be relevant for the efficacy of a certain mAbs. In particular, although listed in Table S1, the effect size score does not consider the reported biological effects on immune cell subpopulations, cytokine levels, blood brain barrier integrity or other readouts of the disease severity. The effect size scores were weighted according to the number of animals using the upper margin in case of unequal group sizes. This weighting method does not take into account that effects of some mAbs were corroborated with results of the respective knockout. In some studies, the mAb was rather used to support the knockout phenotype, which may justify the use of small group sizes. Despite these limitation, the effect size estimate allows for a comparison of outcomes of heterogenous studies and it would be desirable that future studies report the “EAE score vs. time AUCs”, their SD and variances to allow for easier comparison across models and species. 

## 4. Methods

### 4.1. Literature Search, Dosing Estimates and Association Analysis

We systematically searched PubMed for all publications describing controlled studies that investigated effects of monoclonal antibodies in EAE models in laboratory rodents or primates up to August 2017, irrespective of dose, dosing schedule, route of administration and observation time (search terms in Figure 1). Two authors independently assessed each abstract for eligibility and extracted data on characteristics of the experimental model, treatments and outcome measures. We included studies reporting the outcome as either time course of the clinical EAE scores or contingency tables reporting EAE incidences in treatment and control groups. Publications had to state the number of animals per group, route of administration, doses and days of treatment and we excluded reviews, books, letters, clinical trials, case reports, or editorials. We further excluded studies published in non-peer reviewed journals, non-English publications, and studies for which full texts were not available. The following data was extracted and listed in spread sheet columns: article information (title, author, journal, publication year), mAb target, animal species, age, gender, EAE model, immunization or adoptive transfer protocol, dose, time of therapy, route of administration, duration of treatment, clinical EAE scores, statistical methods, molecular or cellular effects and observation time. Studies reporting more than one experiment with different settings were split into 2 or more rows. 

The total doses were normalized per kilogram of body weight and calculated as “single dose” X “number of injections”. For studies which reported doses per animal but not body weight we used body weight estimates of 25 g per mouse and 300 g per Lewis or 200 g per DA rat. 

The effect sizes were calculated according to Cohen’s d (where possible) using reported results of t-tests or Mann Whitney *U*-tests and estimates of the areas under the time courses of the EAE scores. Positive effect sizes were then categorized according to a 6-point scale ranging from 0–6, and meaning no-effect (0), minor (1), mild (2), moderate (3), substantial (4), strong (5) or very strong reduction of the scores (i.e., “6” = 95–100% reduction). For studies, in which the monoclonal antibody increased the clinical scores compared with the control group, that is, where treatment worsened the disease, the effect size was scored as “−1” or “−2”. More severe worsening did not occur. If the effect consisted exclusively in a delay of clinical symptoms without change of disease intensity the effect was considered to be minor, mild or moderate (i.e., 1, 2 or 3) depending on the time of the disease free period. The latter mainly applied to marmoset studies. The effect size score was weighted based on the number of animals per treatment group (*n*), by multiplication of the effect size score with *n*/10. A group size of 10 was used as “standard”, because it is the recommended number of animals per group for EAE studies and was the mean and most frequently used number of animals per group. To assess the associations of effect sizes vs. total doses, vs. single doses or vs. treatment onset, weighted effect size scores were plotted vs. the respective parameters including all experiments irrespective of the target of the mAb, or only those studies which addressed dose responses or onset-dependent responses. Linear regression analyses were used to assess the dependent or independent nature of each two parameters, and were performed for unweighted and weighted effect size scores.

### 4.2. Induction of EAE and Clinical Assessment of the Schedule Dependent Effects of Anti-Itga4

C57BL6/J mice were immunized according to a standard protocol using the Hooke Kit™ MOG35-55/CFA emulsion PTX (EK-2110, Hooke Labs, St Lawrence, MA, USA), which contains 200 µg myelin oligodendrocyte glycoprotein (MOG) 35–55 emulsified in 200 µL Complete Freund’s Adjuvant (CFA). The emulsion was injected subcutaneously at two sites followed by two intraperitoneal (i.p.) injections of 200 ng pertussis toxin (PTX) in phosphate buffered saline (PBS), the first 1–2 h after MOG35-55, and the second 24 h thereafter.

SJL mice were immunized according to a standard protocol using Hooke Kit™ PLP139-151/CFA emulsion PTX (EK-0123), which contains 200 µg myelin proteolipid protein (PLP) 139-151 in 200 µL CFA (Hooke Labs, USA). The emulsion was injected subcutaneously at two sites followed by two i.p. injections of 200 ng PTX in PBS, the first 1–2 h after PLP135-151, and the second 24 h after PLP135–151.

EAE scores and body weights were assessed daily to evaluate the severity and stage of the disease by an observer who was not aware of the treatments. Score 0 means no obvious changes in motor functions. Score 0.5 is a distal paralysis of the tail; score 1 complete tail paralysis; score 1.5 mild paresis of one or both hind legs; score 2 severe paresis of hind legs; score 2.5 complete paralysis of one hind leg; score 3 complete paralysis of both hind legs and score 3.5 complete paralysis of hind legs and paresis of one front leg. Mice reaching scores ≥ 3.5 were euthanized.

Anti-Itga4 monoclonal antibody or placebo was injected i.p. or IV using different schedules and doses in 4 independent experiments in SJL/J or C57BL6 mice each consisting in 10/10 mice for mAb and placebo groups. Experiments were approved by the local Ethic committee for animal research (Darmstadt, Hessen, Germany), adhered to the European and Germany regulations and to the guidelines of GV-SOLAS and were in agreement with ARRIVE.

## Supplementary Materials

Supplementary materials can be found at www.mdpi.com/1422-0067/18/9/1992/s1.
